# Stressful events among adolescents from public schools in a community in Brazil

**DOI:** 10.1192/j.eurpsy.2023.1921

**Published:** 2023-07-19

**Authors:** P. M. Pacheco, M. D. S. Pacheco, D. R. Molini-Avejonas

**Affiliations:** 1 Speech Language Pathology, University of São Paulo, Vila Velha, Brazil

## Abstract

**Introduction:**

Adolescence can be seen as a fundamental stage of life for the construction of the subject, resulting from childhood experiences and decisive for adulthood. It is common stressors to appear during adolescence, due to the lack of necessary resources to deal with a stressful problem or event. In this way, the evaluation of a stressful situation by the adolescent is important, because from it he will develop coping strategies that will help him to deal with the problem.

Stressful life experiences, whether important events or even common annoyances, threaten the adolescent’s well-being, in addition to being linked to mental health and behavior problems, both internalizing ones, such as isolation, somatic complaints and anxiety/depression, as well as externalizing factors such as breaking rules and aggressive behavior. Romantic relationships are related to a major source of stress in the lives of these young people when conflicts, jealousy, aggression and infidelity occur, and have a great impact on the mental health of the individuals involved. Breakups, for example, have been linked to the onset of clinical depression in adolescents.

There are three categories of concern for adolescents: (a) related to achievements, such as success in school or opportunity for success in the future; (b) relationships with colleagues or family members; and (c) social problems such as the environment, poverty and unemployment.

**Objectives:**

To identify and describe stressful life events in adolescents from public schools in a poor community in Brazil.

**Methods:**

The study included 64 adolescents, boys (51.56%) and girls (48.43%), aged between 12 and 16 years old (average 13.76%). The Adolescent Perceived Events Scale (APES) was used, which consists of 90 items that correspond to events that may occur in the adolescent’s daily life.

**Results:**

The most significant stressors for the adolescents in this study were those dealing with the death of close people, such as a friend (100%), a family member (96.29%) and a relative (94.73%). Also appearing as major stressors were “plans that did not work” (91.89%), use of alcohol or drugs by family members (87.5%), loss of a job by parents (75%), imprisonment of a family member (75%), fights with boyfriend or girlfriend (86.66%), breakup (75%) and concern about their own appearance (76.52%).

**Conclusions:**

Events referring to interpersonal relationships were considered the greatest stressors, with percentages above 80%. The present study contributed to the understanding of adolescents’ perceptions of their life events. In this way, we can understand the relationship between stressors and the coping strategies. Furthermore, it allows the proposition of preventive intervention strategies in the school context.

**Disclosure of Interest:**

None Declared

